# Regional and income disparities in treatment and drug adherence of patients with dyslipidemia: a retrospective cohort study in South Korea, 2003–2015

**DOI:** 10.1186/s12877-021-02510-5

**Published:** 2021-10-21

**Authors:** Kyu-Tae Han, Dong-Woo Choi, Seungju Kim

**Affiliations:** 1 Division of Cancer Control and Policy, National Cancer Center, 323 Ilsan-ro, Ilsandong-gu, Goyang-si, 10408 Republic of Korea; 2 Cancer Data Center, National Cancer Center, 323 Ilsan-ro, Ilsandong-gu, Goyang-si, 10408 Republic of Korea; 3 Department of Nursing, College of Nursing, The Catholic University of Korea, 222, Banpo-daero, Seocho-gu, Seoul, 06591 Republic of Korea

**Keywords:** Health disparities, Regional disparities, Socioeconomic status, Dyslipidemia, Statin, Prescription rate

## Abstract

**Backgrounds:**

Health disparities represent a major public health problem that needs to be addressed, and a variety of factors, including geographical location and income, can contribute to these disparities. Although previous studies have suggested that health differs by region and income, evidence on the difference in treatment rate is relatively insufficient. To identify differences in prescription rates by region and income in patients with dyslipidemia.

**Methods:**

Using data from the National Health Insurance Service senior cohort, we included older adults who were diagnosed with dyslipidemia in Korea from 2003 to 2015. Overall prescription rate was determined for patients with dyslipidemia. In addition, medication possession ratio and a defined daily dose were analyzed in patients who were prescribed statins. A generalized estimating equation Poisson model was used to assess differences in prescription rates.

**Results:**

Patients living in rural areas (Chungcheong-do, Jeolla-do, and Gyeongsang-do) had a significantly higher prescription rate than those in metropolitan cities. Unlike the prescription rate, the drug adherence was significantly higher in Seoul, Gyeonggi-do, and Gangwon-do but lower in Jeolla-do and Gyeongsang-do than in metropolitan cities. Patients with low income had lower prescription rates than those with high income, but this difference was not statistically significant.

**Conclusion:**

Our findings demonstrate differences in the treatment rates of patients with dyslipidemia by region and income. Appropriate interventions are needed in vulnerable regions and groups to increase the treatment rate for patients with dyslipidemia.

**Supplementary Information:**

The online version contains supplementary material available at 10.1186/s12877-021-02510-5.

## Introduction

Dyslipidemia, defined as an abnormality in serum cholesterol, is a chronic disease that gradually increases with lifestyle changes and age [1, 2]. Previous studies have suggested that dyslipidemia is a risk factor for other chronic diseases, such as cardiovascular disease (CVD) and cancer. Thus, proper management in patients with dyslipidemia is important to prevent the onset of secondary diseases [3–5]. In particular, early intervention with drugs to lower serum cholesterol such as statins is important [5]. Patients who received early intervention with drugs, such as statins, can lower serum cholesterol levels and decreased the risk of comorbidities.

In 2013, the prevalence of dyslipidemia in Korea was 16.58% and gradually increased with age, especially in women older than 50 years [6]. Age is closely related to serum cholesterol, and with older age, serum low-density lipoprotein levels increase, enhancing the risk of various diseases [2]. Therefore, statins are important for older adults patients with dyslipidemia. In some patients, a low long-term dose has been prescribed due to side effects such as myopathy [7, 8]. Despite these side effects, patients are advised to continue taking the statins because the reduction in the burden of disease associated with lipid-lowering therapy is larger than such effects [9].

Statins are associated with improved survival and reduced risk of CVD [10]. In addition, statins are known to have pleiotropic effects including reduced vascular inflammation, decreased smooth muscle proliferation, and immunomodulation [11–13]. However, despite the importance of lipid-lowering therapy in patients with dyslipidemia, the treatment rate for these patients in Korea is not high, and there are gaps between diagnosis and treatment [10, 11]. In particular, the prevalence of dyslipidemia varies by socioeconomic level and region, possibly resulting in differences that affect the treatment rate [12].

A studies showed that there is geographic inequality in the awareness, treatment, and control of dyslipidemia in patients at middle age and older [13]. Similarly, studies have shown that low-income patients living in urban and rural areas have the lowest awareness and treatment rates [14]. A study found no association between socioeconomic status (SES) and treatment rates in patients with hypertension [15]. Studies have shown that when the access to care is equal, low SES levels do not affect patients’ treatment rate and length of stay [16]. However, in Korea, because most large hospitals are concentrated in metropolitan areas, access to care is different depending on the region. As a result, the treatment rate of dyslipidemia may vary between regions. Although many studies have examined the effect of geographical location and SES on the treatment rate of patients with dyslipidemia, studies those that have focused on statins in older adults are insufficient.

In this study, we evaluated the association of regional and income disparities with the treatment rate of older adults with dyslipidemia. Drug prescription data were analyzed in patients diagnosed with dyslipidemia in Korea from 2003 to 2015. In 2014, insurance standards changed for prescription drugs, which may have affected prescription rates. A subgroup analysis was conducted by year to identify differences in drug prescription rates by region and income due to changes in insurance standards. In addition, we assessed statin dosage and drug adherence to identify differences in treatment rate by region and income.

## Materials and methods

### Database and data collection

This study used Korean National Health Insurance Services cohort data of older adults from 2002 to 2015. Health data was available for 558,147 adults aged 60 years and older from 2002 to 2015 [17]. Theses data were collected from the insurance claims and included demographic information, diagnosis, medications, cost, date of visit, and date of death, if applicable. We analyzed data from patients who received inpatient or outpatient care and were diagnosed as having dyslipidemia based on the International Classification of Diseases, 10th edition (ICD-10; code, E78). From 2002 to 2015, 102,666 patients were treated for dyslipidemia. To focus on patients newly diagnosed with dyslipidemia, we excluded patients who were diagnosed or prescribed drugs for treatment of dyslipidemia (including statin, fibrates, and ezetimibe) before 2002. Next, patients aged 85 years or older were excluded. Finally, we excluded patients who were not followed up year from baseline to 2015 or the year of death or patients over 90 years of age. Ultimately, a total of 85,450 patients and 518,596 cases were included in this study.

### Variables

The variables of interest were region and income status. Because Korea charges insurance premiums according to wealth and income, a patient’s income was measured based on insurance premiums. Premiums ranged from 0 to 10, and we have classified them into four income categories according to quartiles (0–2, low; 3–5, low-moderate; 6–8, moderate-high; 9–10, high). Korea is classified into administrative districts of Seoul, six metropolitan cities, and eight provinces. For this study, regions were classified into seven categories based on administrative districts: Seoul, Gyeonggi-do, Chungcheon-do, Jeolla-do, Gangwon-do, Gyeongsang-do, and metropolitan cities (Incheon, Daejeon, Daegu, Gwangju, Ulsan, and Busan).

The outcome variables were drug prescription rate, drug adherence, and dose in dyslipidemia patients. The dyslipidemia drugs covered by the NHI, namely statins, fibrates, and ezetimibe, were considered in this study (Supplementary Table S1). Patients were evaluated annually on whether they received drug prescriptions after diagnosis of dyslipidemia. First, we classified patients by drug prescription (1 when they were prescribed a drug; 0 when they were not). Second, we evaluated whether patients who were prescribed drugs had a different drug continuity by region and income. To evaluate the continuity of statins in patients who prescribed statin, we calculated the medication possession ratio (MPR) for each patient included in the analysis. We calculated the sum of the total prescription days based on the data for the drug that the patient was prescribed each year during the study period and modified the total supply days so that it did not exceed 365 days. The NHI covered only one drug for each dyslipidemia patient until 2014, when this maximum was expanded to two drugs for those with mixed hyperlipidemia (E782). Therefore, for patients with mixed hyperlipidemia taking two drugs in 2014–2015, the drug they had been taking longer was included in the MPR calculation (Supplementary Table S2). The total prescribed days were divided by 365 days to calculate the annual MPR [18]. Drug adherence was defined as an MPR greater than 80 [19]. Statin dosage was calculated according to the total supply per day and the quantity of the statin, including simvastatin, lovastatin, pravastatin, fluvastatin, atorvastatin, cerivastatin, rosuvastatin, and pitavastatin. From this calculation, we determined the annual cumulative defined daily dose (DDD) for each patient.

Patient demographic data included sex (male, female), age (61–65, 66–70, 71–75, 76–80, and 81–85 years), and the year of dyslipidemia diagnosis (2003–2005, 2006–2010, and 2011–2015). We included each patient’s degree of disability (none, mild, and severe) to assess whether the prescription rate varied by disability. In addition, chronic diseases such as hypertension and diabetes were evaluated based on the ICD-10 code. We evaluated the presence of chronic diseases, including hypertension and diabetes, based on the date of the initial diagnosis of dyslipidemia and classified them into three categories (none, before dyslipidemia diagnosis, and after dyslipidemia diagnosis). Severity was measured by the Charlson Comorbidity Index, and diabetes was excluded from the score. Medical institutions in which patients were treated for dyslipidemia or initially received the diagnosis were classified into four categories (community health center, clinic, hospital, and general hospital).

### Research ethical approval

This study was waiver from the Institutional Review Board of The Catholic University of Korea (IRB number: MC21ENSI0043).

### Statistical analysis

The distribution of each categorical variable was examined by analyzing the frequency and percentage using χ^2^ tests. For continuous variables, t-tests were performed to compare the mean and standard deviation values. Continuous variables were expressed as the means ± standard deviation. The generalized estimating equation (GEE) model was used to account for inter-subject and within-subject correlations [20]. We used the Poisson models for evaluating the association of regional and income disparities with prescription rates. First, we determined the prescription rate in older adults with dyslipidemia, and all variables were entered simultaneously into the fully adjusted model. Second, because the drug reimbursement for dyslipidemia changed in 2014, the difference in prescription rates by region and income was evaluated through subgroup analysis by year (2003–2013, 2014–2015). Third, drug adherence was evaluated based on patients who were prescribed dyslipidemia treatment. The MPR was evaluated for drug adherence higher than 80, and the DDD of statins was used as an outcome variable for the drug dose analysis. All statistical analyses were performed using SAS statistical software version 9.4 (SAS Institute, Cary, NC, USA). A *p*-value less than .05 was considered statistically significant.

## Results

Table 1 shows the baseline characteristics of older adults with dyslipidemia. A total of 85,450 patients were newly diagnosed as having dyslipidemia from 2003 to 2015, of which 46,529 (54.5%) were prescribed a drug for dyslipidemia. By region, the prescription rate was highest in metropolitan areas (*n* = 10,964; 56.2%). In addition, the treatment rate for patients with dyslipidemia did not generally exceed 55% in Seoul, Gyeonggi-do, Chungcheon-do, and Jeolla-do. The prescription rate was the highest in low-income patients (*n* = 10,971; 56.1%) and the lowest in high-income patients (*n* = 16,041; 53.7%). Female patients (*n* = 31,693; 57.4%) were prescribed drugs more often than male patients, and the prescription rate increased each year.

**Table 1:** Baseline characteristics of adults aged 61 years or older with dyslipidemia in South Korea, 2003–2015

	Dyslipidemia treatment			Total n (%)			***p-***value
	Yes n (%)		No n (%)				
**Region**							
Seoul	10,018	(53.3)	8766	(46.7)	18,784	(22.0)	<.0001
Gyeonggi-do	9308	(53.3)	8144	(46.7)	17,452	(20.4)	
Metropolitan	10,964	(56.2)	8542	(43.8)	19,506	(22.8)	
Chungcheon-do	3866	(54.3)	3255	(45.7)	7121	(8.3)	
Jeolla-do	4249	(53.0)	3765	(47.0)	8014	(9.4)	
Gangwon-do	1718	(56.1)	1346	(43.9)	3064	(3.6)	
Gyeongsang-do	6406	(55.7)	5103	(44.3)	11,509	(13.5)	
**Income**							
Low	10,971	(56.1)	8581	(43.9)	19,552	(22.9)	<.0001
Low-moderate	7523	(54.4)	6313	(45.6)	13,836	(16.2)	
Moderate-high	11,994	(54.1)	10,180	(45.9)	22,174	(25.9)	
High	16,041	(53.7)	13,847	(46.3)	29,888	(35.0)	
**Disability**							
None	46,360	(54.5)	38,753	(45.5)	85,113	(99.6)	0.2498
Mild	58	(48.3)	62	(51.7)	120	(0.1)	
Severe	111	(51.2)	106	(48.8)	217	(0.3)	
**Sex**							
Male	13,836	(48.5)	14,696	(51.5)	28,532	(33.4)	<.0001
Female	32,693	(57.4)	24,225	(42.6)	56,918	(66.6)	
**CCI**^a^	1.76	± 1.57	1.86	± 1.65			<.0001
**Diagnosis of diabetes**							
Before dyslipidemia	12,502	(50.5)	12,243	(49.5)	24,745	(29.0)	<.0001
After dyslipidemia	8120	(59.0)	5654	(41.0)	13,774	(16.1)	
None	25,907	(55.2)	21,024	(44.8)	46,931	(54.9)	
**Diagnosis of hypertension**							
Before dyslipidemia	29,669	(54.3)	25,003	(45.7)	54,672	(64.0)	<.0001
After dyslipidemia	7931	(58.9)	5543	(41.1)	13,474	(15.8)	
None	8929	(51.6)	8375	(48.4)	17,304	(20.3)	
**Age**							
61–65	9181	(49.1)	9509	(50.9)	18,690	(10.7)	<.0001
66–70	16,691	(55.2)	13,573	(44.8)	30,264	(19.5)	
71–75	12,741	(55.6)	10,157	(44.4)	22,898	(14.9)	
76–80	6179	(58.5)	4384	(41.5)	10,563	(7.2)	
81–85	1737	(57.2)	1298	(42.8)	3035	(2.0)	
**Hospital**							
Community health center	3431	(58.7)	2417	(41.3)	5848	(6.8)	<.0001
Clinic	27,988	(56.0)	22,004	(44.0)	49,992	(58.5)	
Hospital	4069	(52.3)	3704	(47.7)	7773	(9.1)	
General hospital	11,041	(50.6)	10,796	(49.4)	21,837	(25.6)	
**Year of diagnosis**							
2003–2005	18,603	(49.8)	18,742	(50.2)	37,345	(43.7)	<.0001
2006–2010	21,244	(57.5)	15,733	(42.5)	36,977	(43.3)	
2011–2015	6682	(60.0)	4446	(40.0)	11,128	(13.0)	
**Total**	46,529	(54.5)	38,921	(45.5)	85,450	(100.0)	

^a^Data expressed as mean ± standard deviation

*CCI* Charlson Comorbidity Index

**Fig. 1:**
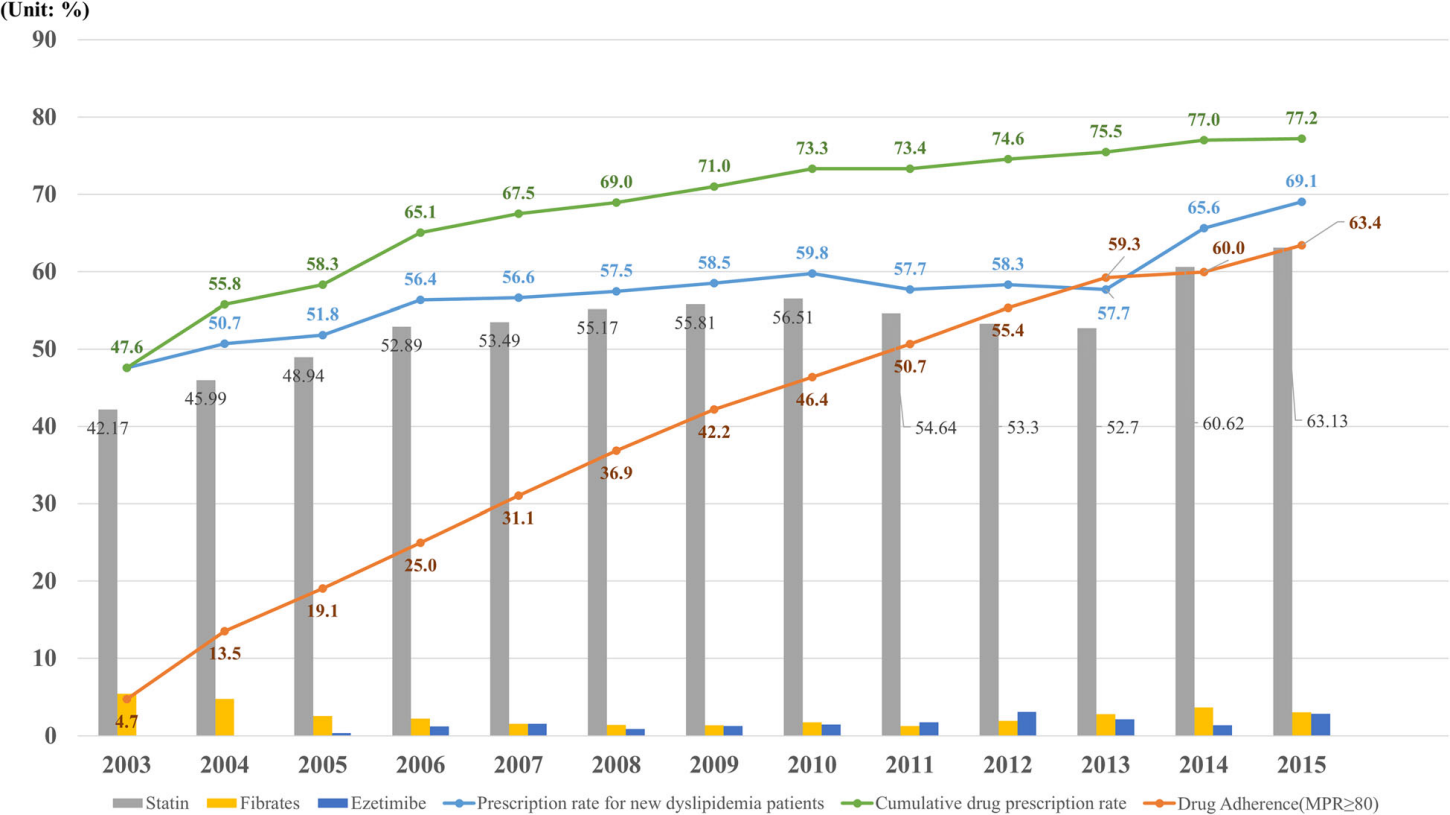
New patient prescription rate, drug type, drug adherence and cumulative prescription rate of dyslipidemia patients in Korea, 2003–2015

Figure 1 shows the prescription rates and cumulative prescription rates for newly diagnosed dyslipidemia patients from 2003 to 2015. The prescription rate was the lowest in 2003 at 47.6% and gradually increased each year until reaching 69.1% in 2015. Cumulative drug adherence (MPR, 80 or higher) of dyslipidemia patients was 4.7% in 2003 but increased to 63.7% in 2015. Most of the patients were prescribed statins, and the prescribing rates for fibrates and ezetimibe were lower.

Supplementary Table S2 shows the MPR in dyslipidemia patients and the cumulative DDD in patients prescribed statins during the study period. Among patients who were prescribed a dyslipidemia drug, 9.7% (*n* = 4510) showed optimal adherence, and the drug adherence was highest among patients living in Seoul (*n* = 788; 7.9%) or high-income patients (*n* = 1155; 7.2%). In addition, the mean MPR was highest among patients living in the Seoul (32.33 ± 26.16) and high income (31.66 ± 25.91). The cumulative DDD was significantly highest in patients living in Gangwon-do (81.80 ± 83.23) and in those with high income (79.80 ± 77.59).

Table 2 shows the results of GEE Poisson regression models for the association of region or income with prescription rate in older adults with dyslipidemia. Patients living in Seoul had a lower drug prescription rate than those living in metropolitan areas, but this difference was not statistically significant (rate ratio [RR], 0.997; 95% confidence interval [CI], 0.993–1.001). Patients living in Chungcheong-do, Jeolla-do, and Gyeongsang-do have a significantly higher drug prescription rates than those living in metropolitan areas. Overall, the drug prescription rate was lower in the low-income group than the high-income group, but this difference was not statistically significant.

**Table 2:** The results of generalized estimating equation Poisson regression models for the association between regional, income and drug prescription in patients with dyslipidemia

	RR	95%CI	
**Region**			
Seoul	0.997	0.993	1.001
Gyeonggi-do	1.003	0.999	1.006
Metropolitan	1.000	–	–
Chungcheon-do	1.021	1.016	1.027
Jeolla-do	1.016	1.011	1.021
Gangwon-do	1.002	0.995	1.010
Gyeongsang-do	1.007	1.003	1.012
**Income**			
Low	1.002	0.999	1.005
Low-moderate	0.999	0.996	1.002
Moderate-high	0.999	0.997	1.002
High	1.000	–	–
**Disability**			
None	1.000	–	–
Mild	1.051	1.018	1.085
Severe	1.003	0.983	1.023
**Sex**			
Male	1.065	1.062	1.068
Female	1.000	–	–
**CCI**	1.003	1.003	1.004
**Diagnosis of diabetes**			
Before dyslipidemia	1.004	1.001	1.007
After dyslipidemia	0.967	0.963	0.970
None	1.000	–	–
**Diagnosis of hypertension**			
Before dyslipidemia	0.958	0.954	0.961
After dyslipidemia	0.946	0.941	0.950
None	1.000	–	–
**Age**			
61–65	1.000	–	–
66–70	0.970	0.966	0.974
71–75	0.957	0.952	0.962
76–80	0.951	0.946	0.956
81–85	0.968	0.962	0.974
**Hospital**			
Community health center	0.983	0.977	0.989
Clinic	0.981	0.978	0.984
Hospital	1.055	1.050	1.061
General hospital	1.000	–	–
**Year of diagnosis**			
2003–2005	1.000	–	–
2006–2010	0.927	0.924	0.930
2011–2015	0.888	0.885	0.891

*RR* rate ratio, *CI* confidence interval, *CCI* Charlson Comorbidity Index

**Fig. 2:**
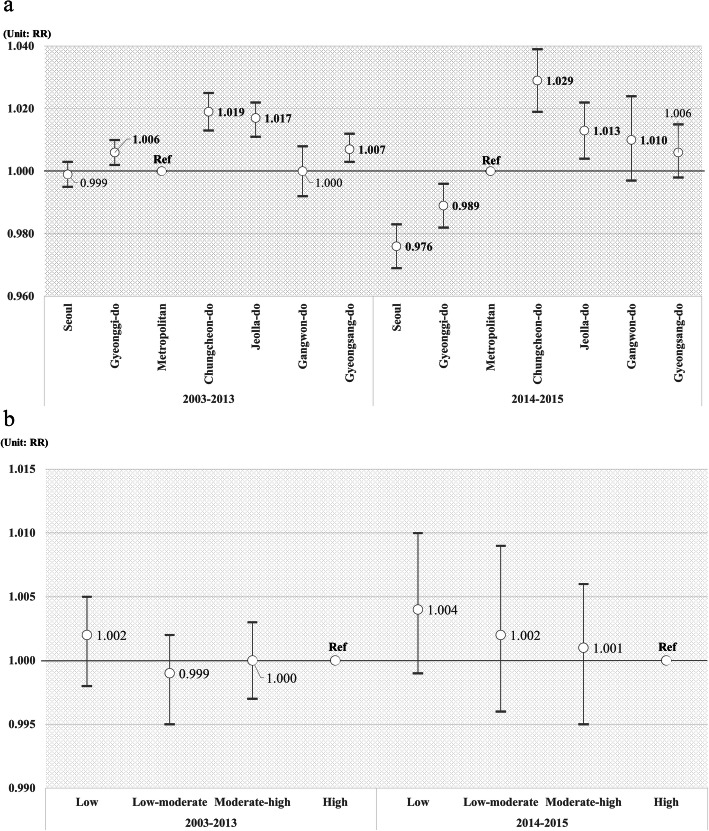
**a** Differences in prescription rates of patients with dyslipidemia in Korea by region. **b** Differences in prescription rates of patients with dyslipidemia in Korea by income

As shown in Fig. 2, the subgroup analyses reveals the differences in prescription rates by region and income. The results of yearly subgroup analysis were similar to the main results. From 2003 to 2013, patients with dyslipidemia in Chungcheong-do, Jeolla-do, and Gyeongsang-do have significantly higher drug prescription rates than those in metropolitan cities. From 2014 to 2015, patients in Seoul (RR, 0.976) and Gyeonggi-do (RR, 0.989) had significantly lower prescription rates, and those in Chungcheong-do (RR, 1.029) and Jeolla-do (RR, 1.013) had significantly higher prescription rates than those in metropolitan areas. No statistically significant results were observed according to income level.

Table 3 shows the regional and income disparity results of the GEE model for drug adherence and statin DDD in patients who were prescribed drugs for dyslipidemia. Compared with metropolitan areas, drug adherence was significantly higher in Seoul (odds ratio [OR], 1.242), Gyeonggi-do (OR, 1.147), and Gangwon-do (OR, 1.058) and significantly lower in Jeolla-do (OR: 0.900) and Gyeongsang-do (OR: 0.935). In addition, drug adherence was significantly lower in low-income groups than in high-income groups. Furthermore, the DDD of statins showed similar results to drug adherence.

**Table 3:** The results of the generalized estimating equation model for the relationship between drug adherence and dose according to region and income

	**OR**	**95% CI**	
**Drug adherence (MPR ≥ 80)**			
**Region**			
Seoul	1.242	1.207	1.278
Gyeonggi-do	1.147	1.115	1.180
Metropolitan	1.000	–	–
Chungcheon-do	0.963	0.926	1.001
Jeolla-do	0.900	0.865	0.935
Gangwon-do	1.058	1.003	1.117
Gyeongsang-do	0.935	0.905	0.967
**Income**			
Low	0.918	0.897	0.940
Low-moderate	0.925	0.901	0.950
Moderate-high	0.948	0.927	0.970
High	1.000	–	–
	**Estimate**	**SE**	***p-*value**
**Defined daily dose (statin)**			
**Region**			
Seoul	10.318	0.833	< 0.0001
Gyeonggi-do	6.734	0.827	< 0.0001
Metropolitan	1.000	–	–
Chungcheon-do	−4.609	1.157	< 0.0001
Jeolla-do	−4.676	1.075	< 0.0001
Gangwon-do	6.469	1.662	< 0.0001
Gyeongsang-do	−4.583	0.921	< 0.0001
**Income**			
Low	−3.894	0.696	< 0.0001
Low-moderate	−3.166	0.748	< 0.0001
Moderate-high	−1.320	0.652	0.0427
High	1.000	–	–

^a^Adjusted for disability, sex, CCI, diagnosis of diabetes or hypertension, age, hospital, and year of diagnosis

*CI* confidence interval, *MPR* medication possession ratio, *OR* odds ratio, *SE* standard error

## Discussion

In Korea, the prevalence of dyslipidemia is gradually increasing, but many patients with the condition remain untreated. In addition, even in the case of patients who received a prescription, awareness of the importance of continuous medication adherence is insufficient. In this study, we found that the treatment rate and drug adherence of dyslipidemia in older adults differed by region and income. In general, the treatment rate of dyslipidemia was higher in the rural areas of Chungcheon-do, Jeolla-do, and Gyeongsang-do than in metropolitan areas. However, unlike drug prescription rates, drug adherence rates were high in Seoul, Gyeonggi-do, and Gangwon-do but rather low in Jeolla-do and Gyeongsang-do. Income level showed a statistically significant association with drug persistence: as income level decreased, drug adherence also decreased. The cumulative DDD showed similar results to drug adherence.

Our study showed that regional and income disparities are present in the treatment of dyslipidemia patients, as well as in the continuity of treatment. These results were similar to those of previous studies showing regional disparities in treatment use for patients with cancer [21] and regional differences in out-of-hospital cardiac arrest incidence and patient outcomes [22]. Other studies have demonstrated that treatment and control rates are low in patients with low-income levels and low education level [14, 23]. However, one study did not identify regional disparities in treatment rate for patients with hypertension [24]. One possible explanation for these differences is that our results were specific to Korean health insurance coverage criteria for dyslipidemia drugs and disparities in access to care.

Korea has a single insurance system, and drugs are covered according to health insurance compensation standards (Supplementary Table S1). Until 2014, the insurance standard for dyslipidemia drugs was based on serum total cholesterol and triglyceride levels, and insurance was applied to one drug for patients with risk factors above a certain level. The need for management based on low-density lipoprotein cholesterol (LDL-C) has been continuously discussed in Korea’s dyslipidemia drug insurance standards, and since 2014, drugs based on serum LDL and triglyceride levels have been applied as insurance benefits. Insurance standards for dyslipidemia affect patient prescriptions, and the number of patients prescribed drugs in the region represents the number of patients who meet insurance standards. Therefore, patients diagnosed with dyslipidemia in regions with low prescription rates may not require medication because their cholesterol has been lowered through continued management. This difference may contribute to the lower prescription rates in regions such as Seoul and increased prescription increases in regions such as Chungcheong-do.

Access to care is an important factor in a patient’s treatment [25], and studies have shown no differences in patient outcomes by SES with equal access to care [16]. In Korea, most medical institutions are concentrated in the capital area, but relatively few hospitals are in rural areas such as Jeolla-do and Chungcheong-do. Differences in access to care can affect patients’ continued treatment, and as shown in our study results, drug adherence is low in regions where the prescription rate is high. A low MPR indicated that patients were prescribed dyslipidemia drugs but did not receive ongoing treatment. Our findings provided evidence for regional disparities in continuity of care and drug prescriptions, suggesting the need to improve access to care to improve treatment rates in areas where continuity of care is not maintained.

Income is an indicator of SES, and although no difference in prescription rates was observed by income, there was a difference in drug adherence. Patients with low income had a lower rate of continuous dyslipidemia treatment than patients with high income. Our findings provide evidence of disparities in vulnerable groups such as older adults with dyslipidemia, suggesting that appropriate care of treatment is needed. To increase drug adherence in older adults with low income, policy makers will need to evaluate existing support for this population and consider additional interventions, accounting for factors that affect health care utilization.

In Korea, indicators related to health disparities are measured under the National Health Promotion Act. However, the health gaps still exist between income groups and regions. These health gaps can lead to the onset of various diseases, and the lack of proper management can lead to increased burden of disease. In particular, changes in prescription rates by region since 2014, when drug insurance standards were changed, suggest that differences in appropriate management exist between regions. Since 2014, the prescription rates for drugs have decreased in capital areas such as Seoul and Gyeonggi-do but increased in areas such as Chungcheong-do. Regions with greater health care access and high drug adherence, and continuous disease management would have lowered the prescription rate by affecting patients’ cholesterol levels. Conversely, regions with increased prescriptions are regions with low drug adherence, and there is a possibility that the number of patients who need prescriptions based on LDL-C levels may increase due to poor management. These results suggest the need for continuous management of dyslipidemia patients and the need for appropriate interventions to reduce the health gap between regions. Policy makers will need to assess and improve the factors that may have these consequences to reduce the health gaps between regions. In addition, health care providers will need to be more actively involved in patient education and management to help patients in vulnerable areas maintain the continuity of care.

Our research findings provide evidence of health disparities according to region and income in Korea, and they are meaningful because a large population of representative older adults was included in our analysis. However, we did not consider clinical factors that could affect patients’ prescription rate, and these unmeasured factors may affect our results. In addition, our findings provide evidence of health disparities in treatment rates between regions, but we did not considered regional factors that could influence these results. Because the drug prescription rate in our study was based on claim data by NHI, prescriptions for drugs not covered by NHI were not included. It is possible that the prescription rate was underestimated, but considering the existing trend, the prescription rate is not expected to be high because the drug prescription rate has not increased significantly since 2014, when the drug standard was changed. Finally, further research will be needed on the various factors that may influence the treatment rate between regions.

## Conclusion

In this study, we found differences in treatment rates for patients with dyslipidemia according to region and income. Our findings suggest that appropriate interventions are needed to reduce this gap in treatment rates between regions. Policy makers will need to increase funding for interventions to reduce health disparities across regions. Moreover, health care providers will need to improve communication to maintain continuity of care with older adults with dyslipidemia.

## Supplementary Information

**Additional file 1.**

## Data Availability

Authors have no rights over the data, NHIS has rights over the data. Data was obtained from NHIS and available with the permission of NHIS.
