# Supplementary material for: Regional and income disparities in treatment and drug adherence of patients with dyslipidemia: a retrospective cohort study in South Korea, 2003–2015
Source: BMC Geriatr. 2021 Oct 21;21:585. doi: 10.1186/s12877-021-02510-5 (PMC8529775; doi:10.1186/s12877-021-02510-5)
Supplement: Supplementary file 1 — Additional file 1. [file 12877_2021_2510_MOESM1_ESM.docx]

**Appendix**

**Supplementary Table S1. Reimbursement standards for dyslipidemia treatment of the National Health Insurance**

In Korea, dyslipidemia drugs are covered by the National Health Insurance, and as of 2014, the reimbursement for drugs has changed.

| ~2013 | 2014.1.1~ |
| --- | --- |
| 1. Hypercholesterolemia  1) No risk factors: Serum total cholesterol level ≥ 250mg/dL  2) Risk factors: Serum total cholesterol level ≥ 220mg/dL  (Patients with coronary artery disease: ≥ 220mg/dL)  3) Drug: HMG-COA reductase inhibitor, bile acid sequestrant, fibrate (Only one drug is accepted) | 1. Hyper Low-density lipoprotein (LDL) cholesterol  1) patients  - Risk factors (0-1): LDL-C ≥ 160mg/dL ; ≥2: LDL-C ≥ 130mg/dL  - Coronary artery disease or disease (peripheral artery disease, abdominal aortic aneurysm, diabetes, etc.): LDL-C ≥ 100mg/dL  - Acute coronary syndrome: LDL-C ≥ 70mg/dL  2) Drug: HMG-COA reductase inhibitor, bile acid sequestrant, fibrate, Niacin, Ezetimibe (Only one drug is accepted) |
| 2. Hypertriglyceridemia  1) No risk factors: Serum triglyceride ≥ 400mg/dL(2 times)  2) Risk factors: Serum triglyceride ≥ 200mg/dL(2 times)  3) Drug: fibrate, Niacin (Only one drug is accepted) | 2. Hypertriglyceridemia  1) Serum triglyceride ≥ 500mg/dL  2) Risk factors: Serum triglyceride ≥ 200mg/dL(2 times)  3) Drug: fibrate, Niacin (Only one drug is accepted) |
| 3. Mixed dyslipidemia (Hypercholesterolemia and Hypertriglyceridemia)  1) No risk factors: Serum total cholesterol level ≥ 250mg/dL and triglyceride ≥ 320mg/dL  2) Risk factors: Serum total cholesterol level ≥ 220mg/dL and triglyceride ≥ 200mg/dL  3) Drug: same as above (Only one drug is accepted) | 3. Mixed dyslipidemia (Hyper LDL-C and Hypertriglyceridemia)  1) Drug: same as above (One of Fibrate or Niacin can be added) |

**B. Dyslipidemia drug prescription status**

| **Supplementary Table S2. Drug adherence of patients treated for dyslipidemia and DDD of statin prescription patients** | | | | | | | | | | | |  |  |  |  |  |
| --- | --- | --- | --- | --- | --- | --- | --- | --- | --- | --- | --- | --- | --- | --- | --- | --- |
|  | | | **Total number of patients  treated for dyslipidemia** | | **Drug Adherence** | | | | | | | **Prescription of statin** | | | | |
|  |  |  |  |  | **MPR(≥80)** | | **MPR** | | | | | **N** | **%** | **DDD** | | |
|  |  |  | **N** | **%** | **N** | **%** | **M** | **SD** | | **p-value** | |  |  | **M** | **SD** | **p-value** |
| **Region** | |  |  |  |  |  |  |  | | |  |  |  |  |  |  |
|  | Seoul | | 10,018 | (21.5) | 788 | (7.9) | 32.33 | ± 26.16 | | | <.0001 | 9,639 | (21.6) | 81.79 | ± 79.29 | <.0001 |
|  | Gyeonggi-do | | 9,308 | (20.0) | 676 | (7.3) | 32.04 | ± 26.01 | | |  | 8,928 | (20.0) | 80.94 | ± 77.71 |  |
|  | Metropolitan | | 10,964 | (23.6) | 614 | (5.6) | 29.49 | ± 24.61 | | |  | 10,522 | (23.5) | 74.98 | ± 74.02 |  |
|  | Chungcheon-do | | 3,866 | (8.3) | 262 | (6.8) | 29.29 | ± 25.91 | | |  | 3,704 | (8.3) | 74.36 | ± 78.63 |  |
|  | Jeolla-do | | 4,249 | (9.1) | 217 | (5.1) | 27.08 | ± 24.37 | | |  | 4,084 | (9.1) | 69.46 | ± 73.25 |  |
|  | Gangwon-do | | 1,718 | (3.7) | 125 | (7.3) | 31.11 | ± 26.28 | | |  | 1,665 | (3.7) | 81.80 | ± 83.23 |  |
|  | Gyeongsang-do | | 6,406 | (13.8) | 371 | (5.8) | 28.96 | ± 24.82 | | |  | 6,163 | (13.8) | 73.00 | ± 72.96 |  |
| **Income** | | |  |  |  |  |  |  | | |  |  |  |  |  |  |
|  | Low | | 10,971 | (23.6) | 702 | (6.4) | 29.59 | ± 25.34 | | | <.0001 | 10,585 | (23.7) | 76.37 | ± 76.43 | <.0001 |
|  | Low-moderate | | 7,523 | (16.2) | 481 | (6.4) | 29.90 | ± 25.43 | | |  | 7,230 | (16.2) | 74.80 | ± 75.15 |  |
|  | Moderate-high | | 11,994 | (25.8) | 715 | (6.0) | 29.63 | ± 24.94 | | |  | 11,453 | (25.6) | 75.44 | ± 76.10 |  |
|  | High | | 16,041 | (34.5) | 1,155 | (7.2) | 31.66 | ± 25.91 | | |  | 15,437 | (34.5) | 79.80 | ± 77.59 |  |
| **Total** | |  | 46,529 | (100.0) | 3,053 | (6.6) |  |  |  | | | 44,705 | (96.1) |  |  |  |

N: number; M: Mean; SD: Standard deviation; MPR: Medication possession ratio; DDD: Defined daily doses

Medication adherence refers to the average number of days that dyslipidemia patients were prescribed medication.

DDD refers to patients who have been prescribed statin among the treatment of dyslipidemia.
